# Proton Radiation-Induced Cognitive Impairment via Disruption of Hippocampal Neuronal Mitophagic Homeostasis

**DOI:** 10.3390/biom16091342

**Published:** 2026-09-15

**Authors:** Longzhen Zhang, Pu Chen, Nan Xu, Junli Chen, Yishu Yin, Wei Liu, Liang Li, Yingying Yu, Weihong Lu, Peng Zang

**Affiliations:** 1 School of Medicine and Health, Harbin Institute of Technology, Harbin 150001, China; 23b936149@stu.hit.edu.cn (L.Z.);; 2 Zhengzhou Advanced Research Institute, Harbin Institute of Technology, Zhengzhou 450001, China; 3 State Key Laboratory of Space Medicine Fundamentals and Application, China Astronaut Research and Training Center, Beijing 100080, China; 4 Institute of Space Environment and Material Science, Harbin Institute of Technology, Harbin 150028, China

**Keywords:** proton radiation, cognitive impairment, mitophagy, apoptosis, BNIP3

## Abstract

**Background:** Proton radiation is both a core component of the space radiation environment and a pivotal modality for precision clinical tumor radiotherapy. Proton exposure can impair cognitive, learning and memory functions, representing a common concern in the fields of deep space aerospace medicine and clinical radiotherapy. Nevertheless, the cellular and molecular regulatory mechanisms underlying proton-induced cognitive impairment remain incompletely elucidated. **Methods:** In vivo and in vitro injury models were established via 100 MeV proton radiation. Specifically, an in vivo model of proton radiation-induced cognitive injury was constructed using C57BL/6J mice (male), and an in vitro radiation injury model was established with HT22 hippocampal neuronal cells to systematically investigate the molecular mechanism of cognitive impairment caused by proton radiation. **Results:** Proton radiation induced a persistent decline in learning and memory capacity in mice, triggered systemic oxidative stress, caused metabolic disturbances of multiple neurotransmitters in the hippocampus, and induced structural mitochondrial damage and decreased mitochondrial membrane potential in hippocampal neurons, suggesting that mitochondria are the key subcellular target of its neurotoxicity. Proton radiation induced oxidative stress in hippocampal neurons. Hyperactivation of mTORC1 inhibited the kinase activity of ULK1 by phosphorylating its Ser757 residue and downregulated ULK1 protein levels, resulting in mitophagy dysfunction and mitophagic flux blockade. Persistent severe oxidative stress prevented damaged mitochondria from being cleared via mitophagy and activated the apoptotic cascade, ultimately leading to hippocampal neuronal death and cognitive impairment. **Conclusions:** This study reveals the molecular mechanism by which proton radiation induces mitophagy dysfunction and hippocampal neuronal apoptosis and subsequently triggers cognitive impairment, providing a new theoretical basis and potential intervention targets for the prevention and treatment of neurocognitive complications associated with space radiation exposure and clinical radiotherapy.

## 1. Introduction

Exposure to proton radiation results in a deterioration of cognitive functions related to learning and memory. Proton radiation exposure to the nervous system generally occurs during deep space exploration and proton radiation therapy for cerebral neoplasms [1,2,3]. In deep space exploration, about 90% of the solar cosmic rays encountered by astronauts are protons, with energy levels spanning from 1 to 1000 MeV, predominantly concentrated in the 10–200 MeV range [4]. Proton radiation therapy for brain cancers utilizes an energy range of 70–250 MeV. The relative biological effectiveness (RBE) for this energy range typically falls between 1.0 and 1.2 [5]. As human deep space travel progresses and proton radiation therapy becomes prevalent in brain tumor treatment, protecting against proton radiation exposure is of paramount importance. While behavioral studies in mouse models have established that proton radiation exposure can induce cognitive damage, direct evidence of pathogenic and physicochemical mechanisms remains limited in current studies [6,7]. The majority of radioprotective pharmaceuticals sanctioned by the U.S. Food and Drug Administration (FDA) concentrate on enhancing survival rates following acute radiation exposure; however, there remain inadequacies in the initial protection and subsequent management of radiation-induced nervous system injury for deep space exploration or radiation therapy.

Gene expression and energy metabolism in cells directly influence cellular fate. Proton radiation induces acute oxidative stress and DNA damage in neuronal cells, leading to sustained production of reactive oxygen species (ROS) and reactive nitrogen species (RNS) in cellular compartments including mitochondria, thereby altering gene expression and cellular energy metabolism. This ultimately results in the apoptosis of neuronal cells and diminished cognitive functions [8]. Mitochondria, being the principal sites of cellular energy metabolism, are among the organelles most profoundly impacted by proton radiation in this process. The nervous system necessitates substantial ATP for functions like signal transmission and the synthesis and transport of neurotransmitters, rendering it one of the tissues with the highest mitochondrial density in the body. Similar to neurodegenerative disorders like Alzheimer’s disease, proton radiation-induced cognitive decline is frequently associated with mitochondrial dysfunction and impaired energy metabolism [9,10,11,12]. Nevertheless, elucidating how proton radiation exposure affects mitochondrial quality in neuronal cells, thereby leading to diminished learning and memory capabilities, presents both conceptual and practical difficulties.

Elucidating the molecular mechanism by which proton radiation induces cognitive impairment is of great significance for the protection against radiation injury in future manned deep space exploration and in proton therapy. Our study revealed that proton radiation caused a persistent decline in learning and memory abilities in mice. Proton radiation induced systemic oxidative stress, as evidenced by decreased total antioxidant capacity, reduced GSH-Px activity, and elevated 8-OHdG levels in mouse serum, along with significant upregulation of pro-inflammatory cytokines such as IFN-γ and IL-1β. These changes were accompanied by metabolic disturbances of neurotransmitters, including GABA, 5-HT, and ACh, in the hippocampus. Furthermore, proton radiation caused structural damage to mitochondria in hippocampal neurons, leading to a decrease in mitochondrial membrane potential, indicating that mitochondria are a key target of proton radiation neurotoxicity. Proton radiation reduced ATP synthesis and increased the AMP/ATP ratio in HT22 cells, thereby activating the energy sensor AMPK. Under physiological conditions, AMPK can activate the mitophagy-initiating kinase ULK1 to initiate ULK1-dependent mitophagy and maintain cellular homeostasis. However, proton radiation induced an increase in ULK1 phosphorylation at Ser757, which suppressed ULK1 activity, ultimately leading to mitophagy dysfunction and mitophagic flux blockade. Under this pathological condition, there was abnormal accumulation of mitophagosomes in hippocampal neurons, along with an increased LC3-II/LC3-I ratio and significantly elevated protein levels of TOM20, p62, and the mitophagy receptor BNIP3. Severe and persistent oxidative stress leads to decompensation of autophagic capacity, whereby damaged mitochondria could not be effectively cleared and continuously release cytochrome c (Cyt c), thereby activating the apoptotic cascade and inducing hippocampal neuronal death, ultimately resulting in cognitive decline.

## 2. Materials and Methods

### 2.1. Key Resources Table

Key resource is shown in Table 1 and Table 2.

### 2.2. Experimental Model and Study Participant Details

#### 2.2.1. Ethical Review

All animal experiments were approved by the Institutional Animal Care and Use Committee (IACUC) of the China Astronaut Research and Training Center (Approval No. ACC-IACUC-2024-021). All procedures were performed in strict accordance with the Guide for the Care and Use of Laboratory Animals (8th edition, National Institutes of Health), the Chinese national standard Laboratory Animal—Guideline for Ethical Review of Animal Welfare (GB/T 35892-2018), and the American Veterinary Medical Association (AVMA) Guidelines for the Euthanasia of Animals. The study was conducted in compliance with the 3R principles (Replacement, Reduction, Refinement) and the ARRIVE guidelines. All efforts were made to minimize animal suffering and distress throughout the experiments.

#### 2.2.2. Animal Treatment

C57BL/6J mice (male, 6–8 weeks old, weighing 18–20 g) were obtained from Nanjing Junke Bioengineering Co., Ltd. All animal procedures were conducted in accordance with the ARRIVE guidelines 2.0. The mice were housed in a controlled environment (20–26 °C, 40–70% humidity) under a 12 h light/dark cycle and divided into three groups (*n* = 8 per group). Prior to the experiment, the animals were allowed to acclimatize for one week. MT, a mitochondria-targeted antioxidant, was administered intraperitoneally at a dose of 2 mg/kg; this group was designated the MT group. The Ctrl and RAD groups received an equivalent volume of normal saline instead of MT. After 21 days of treatment, the mice were anesthetized with isoflurane, placed in a specialized container, and exposed to 100 MeV whole-body proton radiation using a proton/heavy-ion accelerator at a dose of 3 Gy and a dose rate of 3 Gy/h [13]. This dose and dose rate were determined through preliminary experiments conducted in our laboratory and were verified to effectively induce hippocampal neuronal injury and cognitive decline while ensuring animal safety. After proton radiation, MT or normal saline administration was continued for an additional 15 days. The mice were then anesthetized with 0.2 mL of 2.5% tribromoethanol. Once deep anesthesia was achieved, the mice were euthanized and the necessary biological samples were collected for analysis.

#### 2.2.3. Cell Cultures

The mouse hippocampal neuronal cell line (HT22) and HT22 Cell Complete Medium were purchased from Wuhan Procell Life Technology Co., Ltd. The main components of HT22 Cell Complete Medium were DMEM supplemented with 10% FBS and 1% P/S. Cells were trypsinized and passaged when they reached 80–90% confluence. All cells were authenticated by short tandem repeat (STR) profiling upon receipt and were cultured at 37 °C in a humidified atmosphere containing 5% CO_2_. For the MT group, the culture medium was replaced with complete medium containing 2 μM MT 24 h before proton radiation. HT22 cells were irradiated with protons using the same parameters as those described in Section 2.2.2.

### 2.3. Method Details

#### 2.3.1. Behavioral Assessment

Changes in cognitive function in mice were assessed using behavioral tests, including the Y-maze and novel object recognition tests. During behavioral testing, background noise was maintained below 40 dB, and constant air circulation was provided to minimize external interference. For the Y-maze test, each mouse was placed in a Y-shaped Plexiglas apparatus with arms measuring 40 cm in length, 15 cm in width, and 30 cm in height. During a 5 min session, the mouse was allowed to freely explore the maze. Spontaneous alternation was quantified as the percentage of overlapping triplet sets in which the mouse entered three different arms consecutively, relative to the total number of triplet sets. The percentage of spontaneous alternation was calculated as the number of actual alternations divided by the total possible alternations (total number of arm entries − 2) [14], expressed as a percentage:

Spontaneous alternation percentage (SAP) = (number of alternations/(total number of arm entries − 2)) × 100%.

An alternation event was defined as consecutive entry into three distinct Y-maze arms without repeated arm visits. The term (total number of arm entries − 2) represents the theoretical maximum possible alternations for each animal. The novel object recognition test was conducted over two days. On the first day (habituation), each mouse was placed in an empty test box (65 × 45 × 65 cm) and allowed to explore freely for 5 min to become familiar with the environment. On the second day (test day), two identical blue wooden cubes were placed in the same corner of the box, and the mouse was allowed to explore them for 10 min (familiarization phase). Immediately afterward, one cube was replaced with an orange cylinder, and the mouse was returned to the box for a 5 min test. Memory performance was evaluated by calculating the discrimination ratio (DR), defined as the time spent exploring the novel object divided by the total object exploration time [15], expressed as a percentage:

DR (%) = (time exploring novel object/(time exploring novel object + time exploring familiar object)) × 100%

#### 2.3.2. Histology Procedures

Histological examination was performed as previously described [16,17]. Briefly, mouse brain tissue was dissected into blocks of approximately 2–4 mm, fixed in 4% paraformaldehyde at room temperature for 20 min, and then continued to be fixed at 4 °C for a total of approximately 24 h, followed by dehydration, paraffin embedding, and sectioning. For immunofluorescence staining, paraffin-embedded sections were blocked with 5% normal serum in PBS containing 0.3% Triton X-100 for 1 h at room temperature. Sections were then incubated overnight at 4 °C with rabbit anti-TOM20 antibody and mouse anti-LC3B (E5Q2K) monoclonal antibody. After washing, the sections were incubated with Alexa Fluor^®^ 488-conjugated goat anti-rabbit IgG (H + L) and Cyanine 3 (Cy3)-conjugated goat anti-mouse IgG (H+L) for 1 h at room temperature. Nuclei were counterstained with DAPI. Images were acquired using a Zeiss LSM880 confocal microscope (ZEISS, Jena, Germany).

For Nissl staining, brain sections were mounted on gelatin-coated slides. The sections were deparaffinized in xylene, rehydrated through a graded ethanol series to distilled water, and then stained with 0.1% cresyl violet solution for 5–10 min at room temperature. After staining, sections were differentiated in 95% ethanol, dehydrated in absolute ethanol, cleared in xylene, and coverslipped with mounting medium. Images were acquired using a Zeiss Elyra P1 light microscope (ZEISS, Jena, Germany).

For TEM, tissue samples were fixed with 2.5% glutaraldehyde in 0.1 M cacodylate buffer (pH 7.4) overnight at 4 °C and post-fixed with 1% osmium tetroxide for 1 h. After dehydration through a graded ethanol series, samples were embedded in Epon resin. Ultrathin sections (70 nm) were cut with a Leica UC7 ultramicrotome, stained with uranyl acetate and lead citrate, and examined under a JEM-2100Plus (Akishima, Tokyo, Japan) transmission electron microscope (JEOL) operated at 80 kV. Mitochondrial morphology was assessed in at least 10 randomly selected fields per sample.

#### 2.3.3. Molecular Biological Detection

Molecular biological assays were performed as previously described [18,19]. Briefly, for an enzyme-linked immunosorbent assay (ELISA), freshly dissected mouse liver or hippocampal tissue was immediately frozen in liquid nitrogen. The tissue was transferred to a 1.5 mL EP tube, 0.5 mL of pre-cooled PBS was added, and the tissue was ground under liquid nitrogen. The homogenate supernatant was then aliquoted. Whole blood was collected into a procoagulant tube, and serum was separated by centrifugation at 3000 rpm for 10 min at 4 °C. Serum aliquots (0.2 mL) were stored in PCR tubes for subsequent analysis. ELISAs were performed using a Synergy H1 microplate reader (Agilent, Santa Clara, CA, USA) according to the manufacturer’s instructions.

To ensure that RNA and protein were obtained from the same tissue sample, total RNA and protein were extracted from mouse hippocampal tissue using an RNA/Protein Isolation Kit. The extracted RNA was immediately reverse-transcribed into cDNA using a cDNA synthesis kit. qRT-PCR was performed on a CFX Connect Real-Time PCR System (Bio-Rad, Hercules, CA, USA) using SYBR Green premix. The thermal cycling conditions were: 95 °C for 2 min; followed by 40 cycles of 95 °C for 15 s and 60 °C for 15–30 s; melt curve analysis was then performed. Relative gene expression was calculated using the 2^−ΔΔCt^ method with Gapdh as the internal reference.

Protein concentration in hippocampal tissue was determined using the BCA assay. Western blotting was performed using an electrophoresis, transfer, and Western blot kit according to the manufacturer’s protocol.

#### 2.3.4. Proteomics

Frozen hippocampal tissues preserved in liquid nitrogen were lysed in RIPA buffer supplemented with 1× protease inhibitor cocktail and 1× phosphatase inhibitor cocktail. The samples were agitated on ice for 20 min and then centrifuged at 5000× *g* for 20 min at 4 °C. The supernatants were collected, snap-frozen in liquid nitrogen, and stored at −80 °C until analysis. Phosphopeptide enrichment, tandem mass tag (TMT) labeling, and liquid chromatography–tandem mass spectrometry (LC–MS/MS) were performed by Biomarker Technologies Co., Ltd. (Beijing, China).

#### 2.3.5. Mitochondrial Membrane Potential (MMP), ROS and ATP Detection

MMP, ROS, and ATP measurements were performed as previously described [20]. Mitochondrial membrane potential (MMP) was assessed using the JC-1 fluorescent probe. Cells were incubated with JC-1 working solution for 1.5 h at 37 °C in the dark, washed twice with PBS, and immediately imaged using an HIS-SIM system (High Intelligent and Sensitive SIM, CSR Biotech, Guangzhou, China).

Intracellular ROS were measured using the cell-permeant probe 2′,7′-dichlorodihydrofluorescein diacetate (DCFH-DA). HT22 cells grown on coverslips were incubated with 10 μM DCFH-DA in serum-free medium for 30 min at 37 °C in the dark. Cells were then rinsed twice with PBS, fixed with 4% paraformaldehyde, and mounted with DAPI-containing mounting medium. Fluorescence images were acquired using a fluorescence microscope at excitation/emission wavelengths of 488/525 nm.

Cellular ATP levels were measured using the CellTiter-Glo^®^ Luminescent Cell Viability Assay according to the manufacturer’s instructions. HT22 cells were seeded into 96-well white plates and treated as described above. After treatment, the culture medium was removed, and 100 μL of CellTiter-Glo^®^ reagent was added to each well to induce cell lysis. The plates were shaken at room temperature for 10 min, and luminescence was recorded using a microplate reader.

#### 2.3.6. Cellular Immunofluorescence

Immunofluorescence staining was performed as previously described. HT22 cells were seeded onto confocal imaging dishes, fixed with 4% paraformaldehyde for 15 min, permeabilized with 0.1% Triton X-100 for 10 min, and blocked with immunofluorescence blocking solution for 1 h at room temperature. The cells were then incubated overnight at 4 °C with a mixture of rabbit anti-TOM20 (1:200) and mouse anti-LC3B (1:200) primary antibodies. After washing, the cells were incubated with Alexa Fluor^®^ 488-conjugated goat anti-rabbit IgG (H+L) and Cy3-conjugated goat anti-mouse IgG (H+L) (1:500) for 1 h at room temperature in the dark. Nuclei were counterstained with DAPI for 15 min. The cells were mounted with immunofluorescence mounting medium and fluorescence images were acquired using a 60× oil immersion objective on an HIS-SIM system [17].

#### 2.3.7. Live-Cell Imaging

Live-cell imaging was performed using an HIS-SIM system. Briefly, HT22 cells were seeded onto glass-bottom confocal dishes. Mitochondria and lysosomes were labeled with MitoTracker™ Green FM and LysoTracker Red DND-99, respectively. The cells were then imaged on the HIS-SIM system using a 100× oil immersion objective [21,22].

#### 2.3.8. Quantification and Statistical Analysis

Data were plotted and statistical significance was determined using Origin software with the indicated tests. Two-tailed Student’s *t* test, one-way ANOVA, and two-way ANOVA were used to compare differences between groups, as specified in the corresponding figure legends. A *p* value < 0.05 was considered statistically significant. Detailed statistical information for each experiment is provided in the corresponding figure legends.

## 3. Results

### 3.1. Proton Radiation Exposure Causes a Decline in Learning and Memory Abilities in Mice

**Figure 1:**
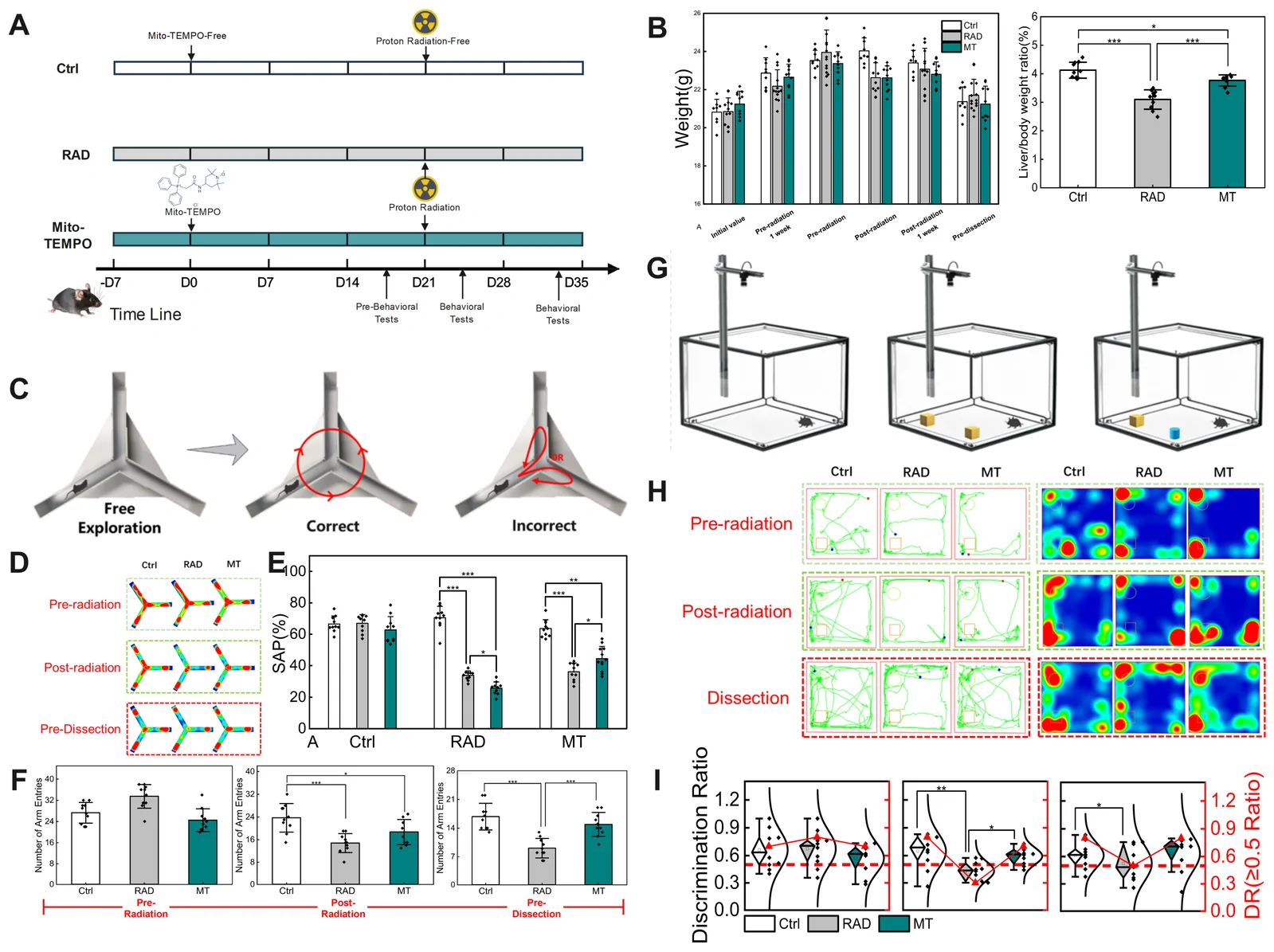
Proton radiation exposure impairs cognitive function in mice through mitochondrial damage in hippocampal neurons. (**A**) Schematic diagram of the mouse experimental process for proton radiation research. (**B**) Body weight changes at different stages and liver/body weight ratios for mice in different groups. (**C**,**D**) Y-maze test: SAP was used to measure cognitive changes, and representative activity heat maps are shown for different groups of mice before radiation, after radiation, and before dissection. (**E**,**F**) Changes in SAP within the same group of mice before radiation, after radiation, and before dissection, as well as changes in SAP among different groups of mice before radiation, after radiation, and before dissection. SAP (%) = (the number of times entering three different arms consecutively/(total number of arms entries − 2)) × 100%. (**G**) Novel object recognition test: Mice first acclimate to an open test arena for 5 min. Then, two cubes of the same color are placed on the same side of the arena, and the mice are allowed to explore for 5 min. One of the cubes is then replaced with a cylinder, and the change in cognitive function is measured by the novel object exploration rate. DR (%) = [time with novel object/(time with novel object + time with familiar object)] × 100%. (**H**) Typical movement trajectories and heat maps of novel object recognition test in mice from different groups before radiation, after radiation, and before dissection. (**I**) Changes in DR of mice in different groups before radiation, after radiation, and before dissection. Data are presented as mean ± SEM. *p* values were calculated by unpaired two-tailed Student’s *t* test or by one-way ANOVA. Data are from three independent experiments (**B**,**E**,**F**,**I**). * *p* < 0.05, ** *p* < 0.01, *** *p* < 0.001.

Given that both neurodegenerative diseases and proton radiation exposure can affect neuronal cells through mitochondrial dysfunction [23], we initially hypothesized that proton radiation exposure could compromise the mitochondria of hippocampal neurons, consequently resulting in a deterioration of cognitive function in mice. To evaluate this notion, we developed a 100 MeV proton radiation mouse model. In the preliminary phase, 4–6-week-old C57BL/6J mice (male) were acclimatized in an SPF-grade animal facility for one week and subsequently randomly allocated to various treatment groups as depicted in Figure 1A for observation. At various time intervals before and after radiation, we monitored the body weight of mice across different groups and subsequently assessed the liver/body weight ratio post-dissection (Figure 1B). In comparison to the Ctrl group, the body weight of mice in the RAD and Mito-TEMPO (MT) groups did not exhibit a significant reduction following proton radiation; however, the liver/body weight ratio demonstrated a significant decline (*p* < 0.001 for RAD and *p* < 0.05 for MT). This reduction in the liver/body weight ratio serves as a critical indicator, implying that proton radiation induced systemic metabolic disorders in mice.

Subsequently, we evaluated the alternations in cognitive behavior of mice following proton radiation by the Y-maze and novel object recognition assessments. In the Y-maze test, we assessed the spontaneous alternation percentage (SAP) of mice at three distinct time intervals: three days before, three days after, and fifteen days (prior to dissection) after proton radiation (Figure 1C). Analysis of mouse behavior (Figure 1D) revealed that the SAP of the Ctrl group mice consistently maintained a high level throughout several testing stages. The SAP of the RAD group mice significantly diminished following proton radiation (*p* < 0.001) and continued to decrease after 15 days (*p* < 0.05). Simultaneously, by quantifying the frequency of mice entering various channels during the observation period in the Y-maze (Figure 1E), we determined that the activity level of mice in the RAD group was markedly lower than that of the Ctrl group at both 3 days and 15 days post-proton radiation (*p* < 0.001). It is significant to note that while the activity level of the MT group mice diminished after 3 days of radiation (*p* < 0.05), it rebounded to the same level as the Ctrl group after 15 days of recovery (Figure 1F). The same time points were used for the novel object recognition test (Figure 1G), wherein the novel object exploration rate (DR) was utilized to evaluate alternations in the cognitive function of the mice. We established a DR value of ≥50% as a criterion for healthy cognitive function. When we analyzed the behavioral trajectories and heat maps of the mice (Figure 1H), we observed that the proportion of mice in each experimental group exhibiting DR ≥ 50% remained elevated at the time point three days before proton radiation. The percentage of mice in the RAD group exhibiting DR ≥ 50% significantly diminished (*p* < 0.01). These findings suggested that proton radiation induced cognitive deficits in mice, potentially linked to oxidative stress within mitochondria.

### 3.2. Cognitive Decline Caused by Proton Radiation Is Mechanistically Linked to Acute Oxidative Stress-Induced Mitophagy in Hippocampal Neurons

**Figure 2:**
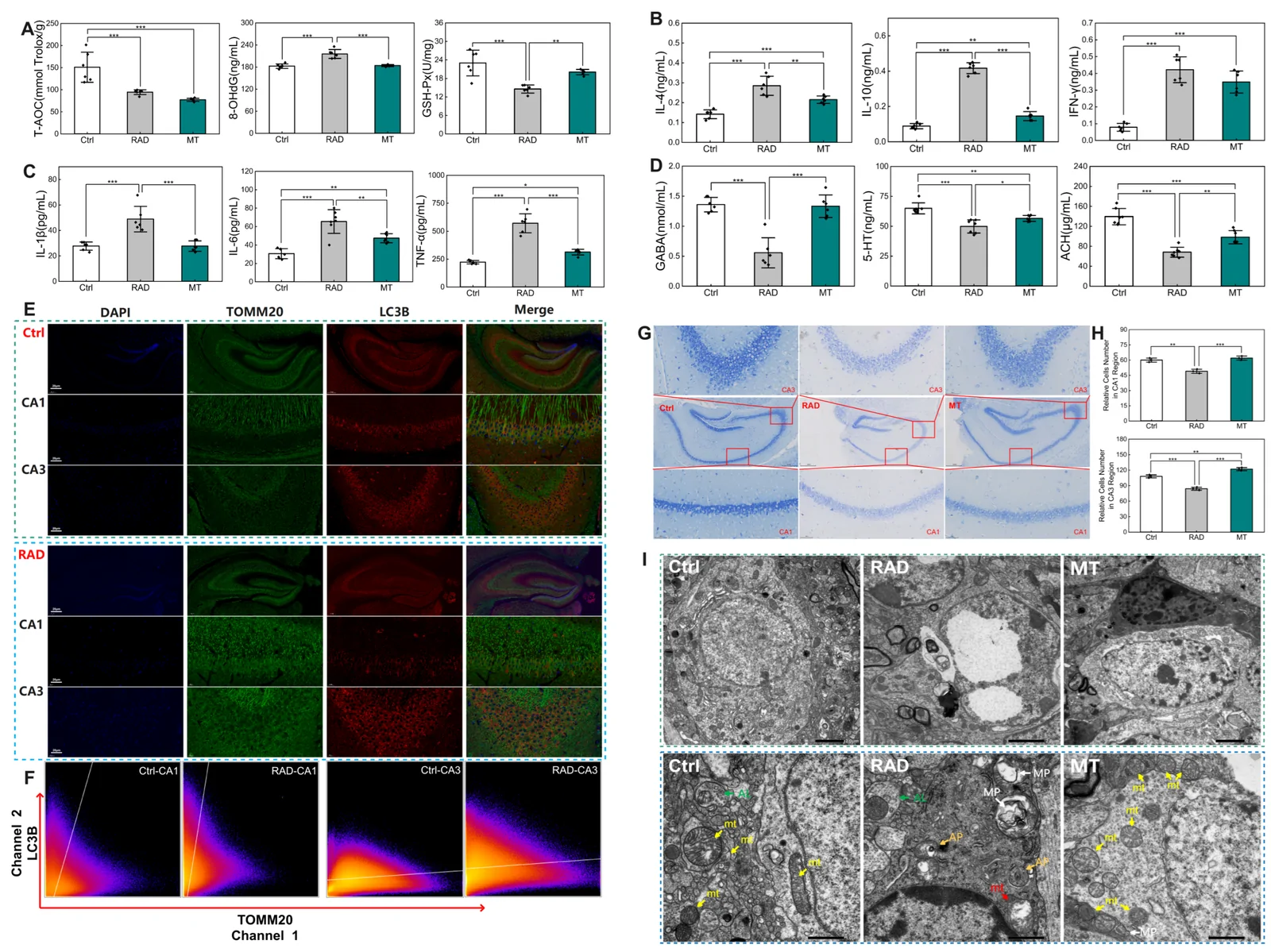
Damage and apoptosis of hippocampal neurons are important pathological mechanisms leading to cognitive dysfunction. (**A**) Measurement of oxidative stress markers in mouse liver tissue by ELISA. (**B**) Measurement of inflammatory factors in mouse serum by ELISA. (**C**) Measurement of inflammatory factors in mouse hippocampal tissue by ELISA. (**D**) Measurement of neurotransmitters in mouse hippocampal tissue by ELISA. (**E**,**F**) Representative immunofluorescence staining images of TOM20 (Alexa Fluor 488) and LC3B (Cyanine 3) in mouse hippocampal tissue, and further observation of the colocalization intensity of TOM20 and LC3B in the CA1 and CA3 regions of the mouse hippocampus. Scale bars: 50 μm and 20 μm. (**G**) Representative Nissl staining images of mouse hippocampal tissue. Scale bars: 50 μm and 20 μm. (**H**) The relative number of Nissl-positive cells in the CA1 and CA3 regions of the mouse hippocampus. (Data are presented as mean ± SEM from three different animal tissues.) (**I**) Representative biological TEM images of mouse hippocampal tissue, where healthy mitochondria are marked in yellow (mt), damaged mitochondria are marked in red (mt), autophagosomes are marked in green (AP), orange APs are considered as active autophagosomes, and mitophagosomes are marked in white (MP). Scale bars: 2 μm and 1 μm. For image panels (**E**,**G**,**I**), *n* = 3 animals. Data in bar graphs (**A**–**D**,**H**) are presented as mean ± SEM (*n* = 6). *p* values were calculated by unpaired two-tailed Student’s *t* test or one-way ANOVA followed by Dunnett’s post hoc test from three independent experiments. * *p* < 0.05, ** *p* < 0.01, *** *p* < 0.001.

Proton radiation can induce an acute oxidative stress cascade in the body [24]. The liver, as the primary center of systemic oxidative metabolism, exhibited a markedly greater expression of antioxidant enzymes and levels of oxidative damage products than other tissues [25,26]. Consequently, we initially measured oxidative stress markers in liver tissue to elucidate the oxidative damage induced by proton radiation in mice (Figure 2A). The findings demonstrated that proton radiation significantly reduced T-AOC and GSH-Px activities in mouse liver, while substantially elevating 8-OHdG levels (*p* < 0.001). This alternation was ascribed to the excessive production of hydroxyl radicals (•OH) induced by proton radiation, resulting in enduring oxidative DNA damage and impairment of antioxidant capabilities [27]. Concurrently, we assessed the inflammatory markers in the serum of mice post-proton radiation (Figure 2B). Proton radiation promotes intracellular DNA double-strand breaks, triggers acute oxidative stress, and activates innate immune cells, including macrophages, dendritic cells, and NK cells, resulting in an imbalance in the Th1/Th2 immune response and a significant elevation of the pro-inflammatory cytokine IFN-γ. Moreover, prolonged oxidative stress promotes a sustained increase in the anti-inflammatory cytokines IL-4 and IL-10 [28]. The administration of MT partially mitigated the effects of proton radiation on the specified oxidative stress markers and inflammatory parameters, indicating that MT may provide a radioprotective effect by safeguarding mitochondria from oxidative stress damage. Hippocampal neuron damage and death are significant pathological pathways contributing to cognitive impairment. To further confirm cognitive impairment in mice induced by proton radiation, we performed biochemical analyses on the hippocampal tissue, liver, and serum of the subjects. The persistent acute oxidative stress induced by proton radiation caused hippocampal neuronal injury and apoptosis, accompanied by significantly elevated pro-inflammatory cytokines IL-1β, IL-6, and TNF-α, as well as markedly decreased neurotransmitters GABA, 5-HT, and ACh in hippocampal tissue (Figure 2C,D).

In response to acute oxidative stress induced by proton radiation, mitochondria in hippocampal neurons clear damaged mitochondria via the mitophagy pathway, thereby preserving normal neuronal energy metabolism [23,29]. We further performed imaging analyses on the hippocampal tissue of mice. Initially, we conducted immunofluorescence labeling of LC3B and TOM20 proteins in mouse hippocampal tissue, concurrently assessing the colocalization of LC3B and TOM20 in the CA1 and CA3 regions (Figure 2E,F). In the CA1 region, the Manders’ tM1/tM2 ratios for LC3B and TOM20 were 0.26 and 0.79, respectively. This suggested that in the Ctrl group, a limited number of mitochondria in the CA1 area were labeled, and the labeled mitochondria exhibited reduced colocalization with LC3B. Conversely, the RAD group showed a substantial increase in mitochondrial labeling. In the CA3 region, the Manders’ tM1/tM2 ratios for LC3B and TOM20 were 2.14 and 4.37, respectively, reflecting a similar tendency to the mitophagy intensity observed in the CA1 region [30,31]. We conducted Nissl staining on mouse hippocampal tissue and subsequently enumerated the Nissl-positive cells in the CA1 and CA3 areas of the hippocampus (Figure 2 G,H). The Nissl staining results of the hippocampal tissue indicated that proton radiation resulted in a disorganized structure of CA1 and CA3 areas of hippocampal neurons in the RAD group, accompanied by a significant reduction in the number of Nissl bodies (*p* < 0.01, *p* < 0.001). We further performed biological transmission electron microscopy (TEM) on mouse hippocampal tissues to directly observe mitophagy occurring in the CA1 region of the hippocampus (Figure 2I). TEM analysis revealed that hippocampal neurons in the control group (Ctrl) exhibited regularly organized cytoplasmic structures with abundant, well-preserved mitochondria displaying distinct and intact cristae. In contrast, neurons in the proton radiation group (RAD) displayed marked cytoplasmic vacuolation, diffusely swollen mitochondria with disrupted and dissolved cristae, and extensive accumulation of autophagosome-like structures within the cytoplasm. These ultrastructural alternations suggested an impairment in the downstream degradative step of mitophagy, whereby autophagosomes sequestering damaged mitochondria fail to be efficiently cleared through the lysosomal pathway. Notably, such morphological changes are characteristic pathological hallmarks of the pre-apoptotic phase. As a mitochondria-targeted antioxidant, MT effectively protected mitochondria from acute oxidative stress damage induced by proton radiation and preserved the normal physiological functions of neurons. Our evidence demonstrates that proton radiation induces acute oxidative stress in the mouse hippocampus, resulting in a significant increase in mitophagosomes within hippocampal neurons. Under physiological conditions, damaged mitochondria are effectively cleared via mitophagy to maintain mitochondrial quality control. However, following proton radiation, mitophagosomes still accumulate abundantly in the hippocampal tissue, ultimately driving hippocampal neuronal apoptosis and leading to cognitive impairment in mice.

### 3.3. Dysregulated Mitophagic Flux Mediates Proton Radiation-Induced Cognitive Deterioration

**Figure 3:**
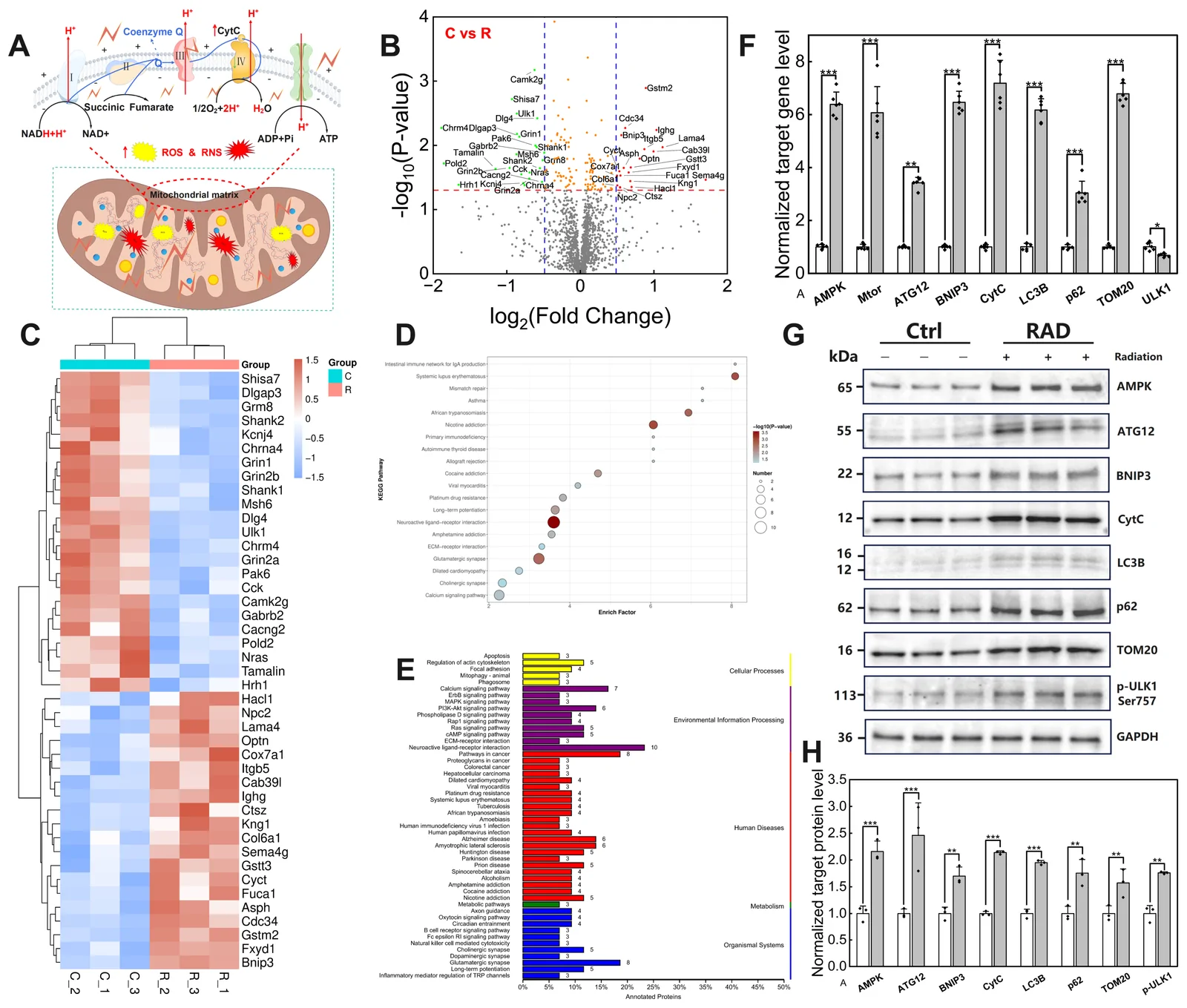
Proteomic analysis of proton radiation-induced mitophagic flux dysregulation in hippocampal neurons of mice. (**A**) Schematic illustration of proton radiation-induced mitochondrial respiratory chain damage. (**B**) Volcano plot of differentially expressed proteins in mouse hippocampal tissue after proton radiation. Thresholds: |log_2_ fold change| > 0.5 and −log10(*p* value) > 1.3. (**C**) Heatmap of differentially expressed proteins in mouse hippocampal tissue after proton radiation. (**D**,**E**) KEGG and GO analysis of differentially expressed proteins in mouse hippocampal tissue after proton radiation. (**F**) Genes encoding differentially expressed proteins in mouse hippocampal tissue after proton radiation. (**G**,**H**) Representative Western blot images and semi-quantitative analysis of differentially expressed proteins after proton radiation. Data are shown as mean ± SEM from three independent animals. Original Western Blot images can be found in Supplementary Materials. *p* values were calculated by unpaired two-tailed Student’s *t* test or by one-way ANOVA followed by Dunnett’s post hoc test, and data are from three independent experiments (**F**,**H**). * *p* < 0.05, ** *p* < 0.01, *** *p* < 0.001.

**Figure 4:**
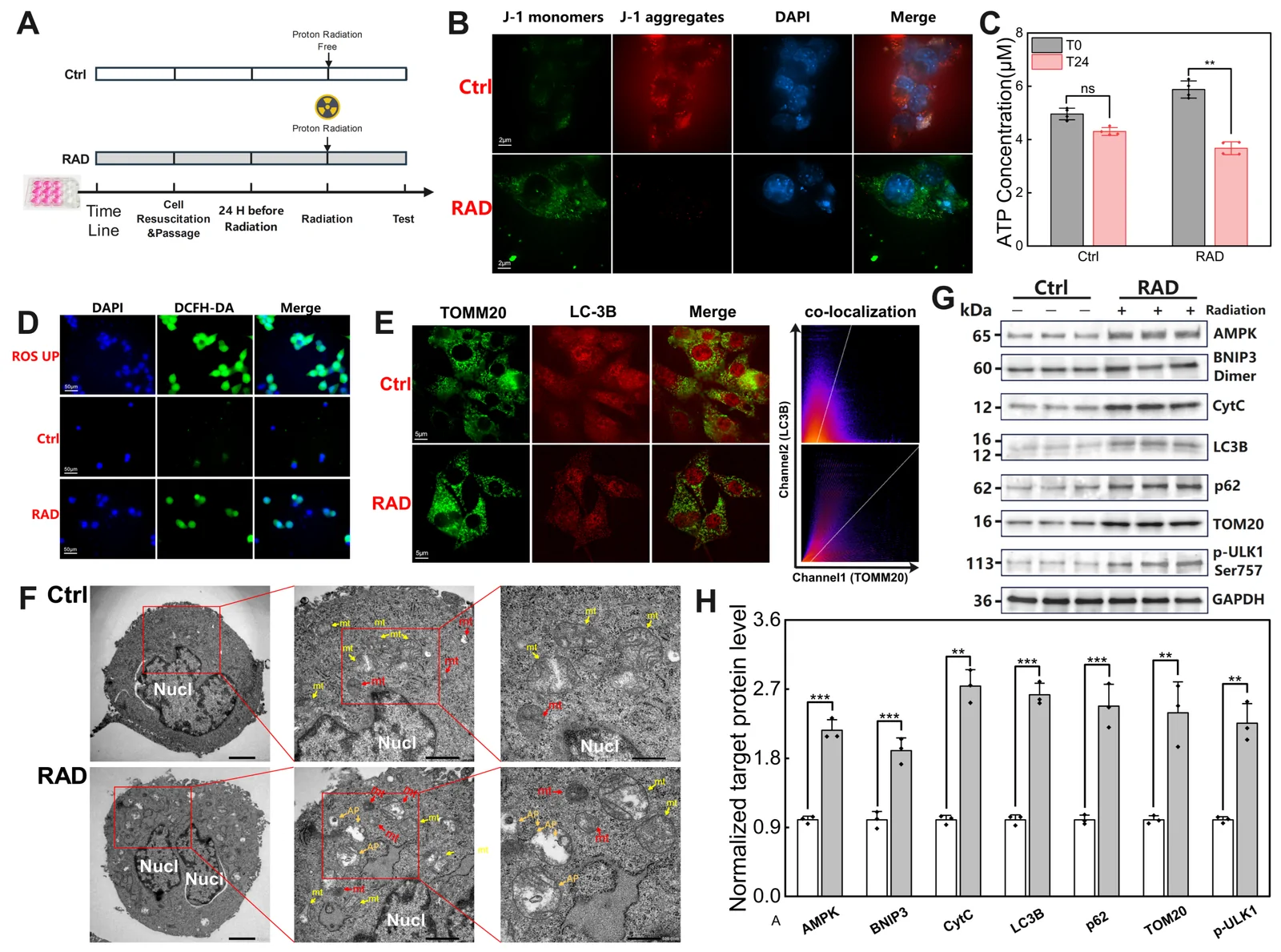
The effect of proton radiation on mouse hippocampal neurons (HT22). (**A**) Schematic diagram of the HT22 experimental process for proton radiation research. (**B**) Representative fluorescence images of mitochondrial membrane potential changes in HT22 cells after proton radiation. JC-1 fluorescence probe loading was performed 4 h after proton radiation, and imaging was conducted using HIS-SIM. Scale bars: 5 μm. (**C**) ATP levels in HT22 cells at 1 h and 24 h after proton radiation, measured using a commercial ATP assay kit and a microplate reader. (**D**) Representative fluorescence images of ROS changes in HT22 cells after proton radiation. After 4 h of proton radiation, the CM-H2DCFDA probe was loaded, and images were taken using a fluorescence microscope. Scale bars: 50 μm. (**E**) Representative immunofluorescence staining images of TOM20 (Alexa Fluor 488) and LC3B (Cyanine 3) in mouse HT22 cells, and colocalization analysis of TOM20 and LC3B in mouse HT22 cells. HT22 cells were fixed 4 h after proton radiation, and imaging was performed using HIS-SIM. Scale bars: 5 μm. (**F**) Representative biological TEM images of mouse HT22 cells. HT22 cells were harvested and fixed with glutaraldehyde at 4 h post-proton irradiation. Color-coded annotations are applied as follows: white labels, “Nucl” for nuclei; yellow labels, “mt” for intact mitochondria; red labels, “mt” for damaged mitochondria; orange labels, “AP” for active autophagosomes; and white labels, “MP” for mitophagosomes. Scale bars: 2 μm, 1 μm, and 500 nm (*n* = 3, respectively, biological replicates). (**G**,**H**) Representative Western blot images and semi-quantitative analysis of differentially expressed proteins in HT22 cells after proton radiation, with data presented as mean ± SEM of three different biological replicates. Original Western Blot images can be found in Supplementary Materials. For imaging experiments (**B**–**F**), images are representative of *n* = 3 biological replicates. Quantitative data in (**C**,**H**) are presented as mean ± SEM from three independent experiments. *p* values were calculated by unpaired two-tailed Student’s *t* test or one-way ANOVA followed by Dunnett’s post hoc test. ** *p* < 0.01, *** *p* < 0.001.

Mitophagic flux dysregulation leads to impaired overall cellular energy metabolism and reduced ATP synthesis efficiency [32]. The nervous system, being one of the highest energy-consuming systems in the body, is particularly vulnerable. When mitophagic flux is blocked in hippocampal neurons, damaged mitochondria accumulate, energy failure ensues, and cognitive impairment manifests (Figure 3A). To obtain an unbiased, system-level view of how proton radiation disrupts hippocampal homeostasis, we performed a quantitative proteomic analysis on hippocampal tissues from Ctrl and RAD mice. In the comparative protein profiling, 24 proteins were downregulated and 22 were upregulated. Notably, cognition-associated scaffolding proteins—including SHANK1, SHANK2, DLGAP3, GRASP, and SHISA7—were consistently reduced (Figure 3B,C), providing a direct molecular correlate for the behavioral deficits observed in Y-maze and novel object recognition tests. Gene Ontology (GO) enrichment analysis of the downregulated proteins further revealed significant representation of dendritic spine organization and ion transport including calcium channels and potassium–chloride cotransporters, indicating that synaptic structural integrity and electrophysiological responsiveness are primary casualties of proton radiation. Crucially, the upregulated proteins painted a remarkably different but mechanistically linked picture. They were overwhelmingly assigned to mitochondrial compartments (inner membrane, respiratory chain complex II, mitochondrial translation), confirming that mitochondria are the primary subcellular targets of proton radiation. Simultaneously, we observed a substantial enrichment of autophagosomal membrane proteins—a finding that, at first glance, might suggest enhanced autophagy. However, when interpreted in the context of our ultrastructural (Figure 2I and Figure 4F) and biochemical (LC3-II/p62 accumulation) data, collectively, this observed accumulation does not denote successful degradative processing; instead, it is consistent with defective autophagosomal elimination, consistent with a blockage in mitophagic flux. The co-accumulation of mitochondrial constituents and autophagosomal markers within the same proteomic dataset is the hallmark of a stalled quality control system. In this context, damaged mitochondria are effectively tagged and sequestered, indicating that autophagosome biogenesis and cargo recruitment remain operational; however, their failure to be delivered to lysosomes for terminal degradation reveals that the late-stage degradative step is compromised. KEGG pathway analysis (Figure 3C–E) further corroborated this pathological cascade. Pathways related to phagosome and lysosome were enriched, but this should be interpreted as a compensatory upregulation of the clearance machinery that ultimately proves insufficient, consistent with the elevated BNIP3 and LC3-II levels we observed by Western blot. Concurrently, the apoptosis pathway was significantly enriched, indicating that the persistent retention of damaged mitochondria triggers the intrinsic death cascade, as evidenced by elevated cytosolic cytochrome c. Intriguingly, pro-survival pathways such as PI3K–Akt and MAPK were also activated, suggesting a futile compensatory anti-apoptotic attempt that is eventually overwhelmed. Moreover, extensive dysregulation of neuroactive ligand–receptor interaction, glutamatergic synapse, dopaminergic synapse, and cholinergic synapse pathways directly implicate synaptic transmission deficits in memory impairment. The additional enrichment of calcium/cAMP signaling and actin cytoskeleton dysregulation further underscores how mitochondrial energy failure secondarily destabilizes synaptic structure and plasticity. Beyond synapse-centric pathways, our proteomic data also revealed alternations in endoplasmic reticulum stress, lipid metabolism, and neurodevelopmental pathways, which may act as auxiliary pathological amplifiers. However, the central, rate-limiting event that connects all these disturbances is the mTORC1-ULK1 axis dysfunction: the downregulation of ULK1 cripple’s autophagosome initiation, while the compensatory upregulation of BNIP3, though aimed at recruiting more damaged mitochondria, is insufficient to overcome the initiation bottleneck. This uncoupling is manifested as an upregulated cargo receptor alongside a downregulated initiation kinase, establishing a rate-limiting barrier to mitophagic clearance. Damaged mitochondria are increasingly sequestered, yet the degradative machinery itself is compromised. Consequently, a process that initially serves as an adaptive stress defense progressively devolves into a maladaptive pro-apoptotic cascade, culminating in hippocampal neuron loss and cognitive decline.

The mRNA expression, protein expression, and phosphorylation levels of target molecules in the hippocampal tissue of mice post-proton radiation were assessed using quantitative reverse transcription polymerase chain reaction (qRT-PCR) and Western blot techniques. All primers used in this study are listed in Table 1. The findings indicated that after proton radiation exposure, the mitophagy pathway in hippocampal neurons exhibited significant disorder and imbalance: the mRNA and total protein levels of the core mitophagy initiation kinase ULK1 were markedly downregulated, while the mitophagy receptor BNIP3 was compensatorily upregulated. The opposite changes in these two suggested that the initiation step of mitophagy is obstructed, leading to decreased clearance efficiency of damaged mitochondria and their abnormal accumulation within the cell.

Proton radiation-induced acute oxidative stress and disturbed growth-factor signaling may promote mTORC1-dependent inhibitory phosphorylation of ULK1 at Ser757, which could contribute to reduced ULK1 activity. Of note, this phosphorylation event might also arise from alternative kinase/phosphatase pathways, independent of mTORC1 signaling. Proton radiation can continuously activate mTORC1 by activating the PI3K/Akt pathway. The activated mTORC1 directly phosphorylates the Ser757 site of ULK1, which on one hand blocks the binding of ULK1 to AMPK, inhibiting its kinase activity, and on the other hand can trigger the ubiquitin-mediated degradation of ULK1. Mechanistically, mitochondrial dysfunction impairs ATP production and elevates the AMP/ATP ratio, thereby activating the energy sensor AMPK, which in turn directly phosphorylates ULK1 at Ser317 and Ser777 to initiate mitophagy [33,34,35]. Proton radiation-induced excessive ROS can oxidatively modify the AMPK kinase domain, leading to its functional inactivation, preventing it from providing protective activation and stabilization to ULK1, ultimately resulting in the dominant inhibitory effect of mTORC1.

Although the downregulation of ULK1 expression hinders the initiation of mitophagy, oxidative stress induced by mitochondrial damage can compensatorily upregulate the mRNA and protein levels of BNIP3 through bypass transcriptional pathways such as FOXO3a and HIF-1α, promoting its localization and enrichment on the outer mitochondrial membrane. This is a defensive compensatory response of the cell to mitochondrial damage. Cells attempt to recruit more damaged mitochondria into the autophagic degradation pathway by increasing receptor abundance [36,37]. However, due to insufficient expression of ULK1, the nucleation and elongation stages of autophagosomes are hindered, preventing the downstream autophagic execution system from functioning effectively. Although there is a certain degree of stress-induced increase in the expression of ATG12, it can combine with ATG5 and ATG16L1 to form the ATG12–ATG5–ATG16L1 complex with the assistance of the E1-like enzyme ATG7 and the E2-like enzyme ATG10 and catalyze the lipidation modification of microtubule-associated protein 1 light chain 3 (LC3-I) to generate LC3-II [38,39,40]. The detection results showed that the proportion of LC3-II increased from 32.24 ± 9.61% in the Ctrl group to 57.49 ± 5.12% in the RAD group. However, this increase was not due to the activation of the autophagic pathway but rather an accumulation effect attributable to impaired late-stage degradation causing mitophagic flux dysregulation, whereby LC3-II could not be effectively degraded by lysosomes. At the same time, p62, a classic marker of mitophagic flux, could not be effectively cleared and accumulated intracellularly, further confirming the significant decrease in mitophagic flux attributable to impaired late-stage degradation, which was consistent with the downregulation of ULK1 and insufficient mitophagy initiation [13]. The disruption of the mitophagy pathway directly leads to the inability to timely clear damaged mitochondria, resulting in the continuous accumulation of functionally abnormal mitochondria within the cell. In the early stages of damage, BNIP3-mediated compensatory mitophagy can partially clear mildly damaged mitochondria, limiting the release of Cyt c. However, as ULK1 continues to downregulate and mitophagy efficiency declines, the integrity of the outer membrane of damaged mitochondria deteriorates, leading to continuous leakage of Cyt-c into the cytoplasm. This ultimately activates the intrinsic apoptotic pathway, inducing the death of hippocampal neurons and forming an important molecular basis for proton radiation-induced cognitive function impairment [41,42].

### 3.4. Proton Radiation-Induced Mitophagic Flux Dysregulation Leads to HT22 Neuronal Death

To explain the mechanism of proton radiation-induced damage to mouse hippocampal tissue, we constructed a mouse hippocampal neuron (HT22) damage model generated by proton radiation (Figure 4A). Following proton radiation, the JC-1 fluorescent probe was utilized to assess the mitochondrial membrane potential of HT22 cells. Proton radiation induced acute oxidative stress that impaired the mitochondrial respiratory chain, elevated mitochondrial membrane permeability, and markedly reduced the mitochondrial membrane potential (ΔΨm) (Figure 4B). Mitochondria, the primary sites of cellular energy metabolism, are highly sensitive to proton radiation. In HT22 cells, intracellular ATP levels exhibited a transient increase followed by a continuous decline after radiation. Immediately after radiation (T0), mitochondria mounted a stress-induced compensatory response, temporarily enhancing ATP synthesis. However, as the mitochondrial membrane potential (ΔΨm) progressively declined, mitochondrial function became irreversibly compromised, leading to the selective removal of damaged mitochondria via mitophagy. Consequently, intracellular ATP levels decreased markedly 24 h after radiation (Figure 4C). We employed the fluorescent probe DCFH-DA to assess alternations in ROS levels in HT22 cells. In comparison to the Ctrl group, ROS levels in HT22 cells were markedly elevated following proton radiation, primarily attributable to proton radiation inducing mitochondrial oxidative stress and impairing mitochondrial DNA (Figure 4D). Following proton radiation, ROS levels increased in HT22 cells, mitochondrial respiratory chain function was impaired, and ΔΨm decreased, thereby inhibiting ATP synthesis and increasing the AMP/ATP ratio, which in turn activated AMPK and induced mitophagy in HT22 cells to clear damaged mitochondria [43,44,45]. This explains the compensatory increase in ATP content. We proceeded with immunofluorescence labeling of TOM20 and LC3B in HT22 cells and conducted a statistical analysis of the colocalization extent of TOM20 and LC3B [30,31] (Figure 4E). The results showed that only 17.0% of the LC3 signals in the control group HT22 cells co-localized with the mitochondrial marker TOM20, indicating that the basal mitophagy in hippocampal neurons is maintained at a low steady state under physiological conditions. After proton radiation, the co-localization ratio of LC3 and TOM20 in HT22 cells significantly increased to 36.3%, and the number of autophagosomes containing mitochondria significantly increased, suggesting abnormal blockage of mitophagic flux. The results of TEM observation of the ultrastructure of HT22 cells (Figure 4F) showed that the mitochondria in the control group cells were intact in morphology, with clear and regular cristae structures. In contrast, the mitochondria in the cells after proton radiation generally exhibited swelling, cristae rupture, and dissolution, along with the accumulation of numerous autophagosome-like structures containing mitochondria in the cytoplasm. In summary, we have preliminarily observed at the cellular level that proton radiation significantly increases the number of damaged mitochondria in HT22 cells, with a blockage in the upstream and downstream processes of mitophagic flux, leading to the abnormal accumulation of autophagosomes containing damaged mitochondria within the cells. Damaged mitochondria could not be effectively cleared through the autophagic pathway, leading to an imbalance in the mitochondrial quality control system, which in turn disrupts the overall metabolic homeostasis of the cell. Meanwhile, damaged mitochondria continuously release Cyt c into the cytoplasm, activating the intrinsic apoptotic cascade and ultimately inducing apoptosis in HT22 cells.

To dynamically track the real-time process of proton radiation-induced mitophagy in HT22 cells, we used Mito-Tracker and Lyso-Tracker to label mitochondria and lysosomes, respectively. We observed the dynamic process of autophagosome–lysosome fusion and degradation in the mitophagy pathway, continuously imaging live cells in real-time 8–12 h post-proton radiation (Videos S1, S2). The results showed that in the control group (Ctrl), the mitochondria in HT22 cells were mostly healthy, tubular, or rod-shaped, with intact morphology and active metabolism. Under physiological conditions, cells maintained a low level of functional mitophagy, with lysosomes normally encapsulating and degrading a small number of damaged mitochondria, thereby maintaining mitochondrial homeostasis. In the RAD group, the mitochondria within HT22 cells exhibited damage such as shrinkage and excessive fission, while the number of lysosomes increased compensatorily and clustered around the damaged mitochondria. This phenotype suggested that cells initiated a clearance response to the large number of damaged mitochondria induced by radiation, but the lysosomes were unable to effectively complete the final degradation, ultimately resulting in a blockage in the downstream stages of mitophagy.

After proton radiation, the detection of mitophagy-related proteins in HT22 cells indicated that the protein levels of p-ULK1 (Ser757), AMPK, BNIP3, LC3B, p62, TOM20, and Cyt c in HT22 hippocampal neurons were significantly upregulated, fully demonstrating the molecular characteristics of mitophagy dysfunction induced by proton radiation, ultimately triggering cell apoptosis: among them, the elevated level of p-ULK1 (Ser757) suggested that mTORC1, by phosphorylating the Ser757 inhibitory site of ULK1, weakened the initiation capability of mitophagy, while the upregulation of AMPK and the mitophagy receptor BNIP3 was a compensatory stress response of the cells to mitochondrial damage, but its effect is insufficient to reverse the inhibitory trend of mitophagy initiation; the simultaneous increase in LC3B and the autophagic substrate p62 further confirmed the blockage of the downstream degradation process of mitophagic flux, leading to the accumulation of autophagosomes containing damaged mitochondria within the cells, which could not be effectively cleared through the lysosomal pathway; the increase in the mitochondrial outer membrane marker TOM20 further indicated that the damaged mitochondria failed to achieve quality control through mitophagy, leading to their massive accumulation within the cells; and the significant rise in Cyt c levels in the cytoplasm suggested that the integrity of the mitochondrial membrane had been compromised, triggering the intrinsic apoptotic cascade.

## 4. Discussion

Proton radiation, the predominant high-energy particle in the space radiation environment, is also a commonly employed modality in tumor radiotherapy; its detrimental effects on the central nervous system upon exposure therefore warrant increased scrutiny. Prior research has established that proton radiation induces DNA damage, protein oxidation, and lipid peroxidation through direct ionization and indirect free radical generation, leading to diminished cognitive function. However, the molecular mechanisms by which proton radiation causes cognitive impairment remain poorly understood [10,46,47,48,49]. This study systematically investigated the molecular pathways by which proton radiation induces cognitive impairment, employing both a mouse model exposed to 100 MeV proton radiation and an in vitro HT22 hippocampal neuron model.

Our results first demonstrated that proton radiation can cause a sustained decline in learning and memory capabilities in mice, confirming its enduring neurotoxic effects on the central nervous system [6,7,50,51]. Proton radiation induced systemic oxidative stress, as evidenced by reduced total antioxidant capacity in the liver, decreased GSH-Px activity, and elevated 8-OHdG levels, along with significantly upregulated pro-inflammatory factors such as IFN-γ and IL-1β in both serum and hippocampal tissue. Notably, oxidative stress metabolites in the liver can indirectly influence the nervous system via the “liver–brain axis,” while inflammatory mediators in the bloodstream can also activate neural pathways through the circulatory system, thereby intensifying the effects of proton radiation on cognitive function. These changes were associated with metabolic dysregulation of neurotransmitters, including GABA, 5-HT, and ACh, in the hippocampus. Proton radiation can activate central neuroinflammatory cascades through dual mechanisms: direct ionizing damage and indirect oxidative stress. Radiation stimulation promotes the massive generation of ROS and RNS in the hippocampus, inducing lipid peroxidation, protein denaturation, and DNA double-strand breaks. These injuries activate the DNA damage response (DDR) pathway, which, together with oxidative stress signals, activates the nuclear factor kappa-B (NF-κB) pathway, directly initiating the gene transcription of pro-inflammatory cytokines such as IL-1β, IL-6, and TNF-α [52,53]. Meanwhile, proton radiation induces rapid polarization of resting central microglia toward a pro-inflammatory phenotype, enhancing their phagocytic activity and promoting substantial secretion of pro-inflammatory cytokines and chemokines. Persistently activated microglia can sustain chronic central neuroinflammation for up to several months, representing a core pathological mechanism mediating long-term radiation-induced cognitive decline [54]. Proton radiation-induced oxidative stress generates ROS that can directly oxidize and impede the function of glutamic acid decarboxylase (GAD), hence obstructing the synthesis of GABA. The synthesis and metabolism of 5-HT are modulated by oxidative stress and inflammation. The function of its rate-limiting enzyme, tryptophan hydroxylase (TPH), is contingent upon tetrahydrobiopterin (BH4) and mitochondria-derived ATP. The production of acetylcholine (ACh) is significantly reliant on acetyl-CoA produced by mitochondria, and mitochondrial failure directly results in a reduction in ACh levels. 5-HT and ACh, key neurotransmitters regulating cognition and memory, rely on sufficient ATP supply to maintain optimal synaptic transmission efficiency; mitochondrial dysfunction may therefore diminish overall neuronal transmission efficacy, further exacerbating cognitive impairment [55]. Collectively, these data provide biochemical evidence that proton radiation leads to cognitive and behavioral deterioration [27,28,55,56]. Moreover, proton radiation caused damage to the mitochondrial architecture of hippocampal neurons, resulting in reduced membrane potential and respiratory chain impairment [46,57], unequivocally demonstrating that mitochondria are a primary target of proton radiation neurotoxicity.

Mitophagy dysfunction is a core pathological link in proton radiation-induced hippocampal neuron damage and cognitive impairment. Under physiological conditions, mitophagy maintains mitochondrial quality control and intracellular homeostasis by selectively removing damaged mitochondria; however, proton radiation can cause a significant accumulation of damaged mitochondria within the hippocampal tissue, ultimately inducing hippocampal neuron apoptosis and mediating cognitive impairment. This study confirmed that proton radiation can lead to the abnormal accumulation of autophagosomes containing damaged mitochondria within hippocampal neurons, manifested as an increased LC3-II/LC3-I ratio and significant upregulation of TOM20, p62, and the mitophagy receptor BNIP3 protein levels. This process is primarily regulated by the BNIP3-mediated mitophagy pathway, a receptor-dependent and ubiquitination-independent mitophagy mechanism distinct from the canonical PINK1/Parkin-dependent pathway [13,58,59]. Proton radiation-induced oxidative stress damage downregulates ULK1 total protein expression on one hand, and on the other hand, it inhibits its kinase activity by promoting phosphorylation at the Ser757 site. Together, these two mechanisms jointly interfere with the initial regulatory stage of mitophagy; meanwhile, the downstream autophagolysosomal degradation pathway becomes blocked, preventing the normal completion of autophagic flux. The abnormalities at both levels together result in a comprehensive disorder of mitophagy function [60,61]. When mitochondrial damage is mild, mitophagy plays a protective role by removing damaged mitochondria and blocking Cyt c release. However, under the sustained severe damage caused by proton radiation, mitophagy function undergoes irreversible disruption, and damaged mitochondria cannot be effectively cleared. This leads to continuous Cyt c release, activation of the apoptosis cascade, and ultimately results in progressive loss of hippocampal neurons and cognitive decline [42,58,62]. In addition to mitochondrial–autophagic and inflammatory injury, ionizing-radiation-mediated radiomodulation, which modulates neuronal membrane physiology and can suppress pathological neuronal activity in clinical contexts, may represent a complementary biological mechanism participating in proton-radiation-induced brain dysfunction [63]. This study establishes a close link between mitochondrial quality control and cognitive function, providing a new theoretical framework for prevention strategies against cognitive impairment induced by proton radiation.

## 5. Conclusions

Proton radiation, a major component of space radiation and a widely used radiotherapy modality, exerts long-lasting neurotoxic effects and triggers persistent cognitive deficits. In this work, using a 100 MeV proton-irradiated mouse model and HT22 hippocampal neuron model, we systematically delineated the multi-layered molecular mechanisms underlying proton radiation-evoked cognitive impairment. Proton exposure elicits systemic oxidative stress and peripheral inflammation, which can propagate neurotoxic signals via the liver–brain axis and circulation to disturb hippocampal neurotransmitter homeostasis. At the central nervous system level, radiation-initiated ROS/RNS burst activates the DDR and NF-κB signaling cascades, driving microglial polarization toward a pro-inflammatory phenotype and sustaining chronic neuroinflammation. Concurrently, oxidative stress and mitochondrial structural/functional damage disrupt the synthesis of GABA, 5-HT and ACh, impair synaptic transmission, and further compromise cognitive performance. Our findings further identify defective BNIP3-dependent mitophagy as a core pathological driver. Proton radiation suppresses ULK1 activity and blocks mitophagic flux, preventing the clearance of injured mitochondria. Accumulation of damaged mitochondria promotes cytochrome c release and neuronal apoptosis, ultimately mediating progressive cognitive decline. Beyond oxidative stress, neuroinflammation and mitophagy failure, radiation-induced modulation of neuronal membrane physiology may also contribute to brain dysfunction. Collectively, this study clarifies the causal chain connecting mitochondrial quality control disruption and cognitive impairment upon proton radiation. These results advance the mechanistic understanding of space proton neurotoxicity and offer novel theoretical foundations for developing preventive interventions against radiation-associated cognitive deficits.

### Limitations of the Study

This study has several notable limitations that should be acknowledged. First, all experiments were performed at a single proton dose with a fixed dose rate. Given that mitophagy typically exhibits a biphasic, dose-dependent response to ionizing radiation, varying doses and dose rates of proton exposure may differentially modulate mitophagic activity and the trajectory of cognitive decline; conclusions from the current setting cannot be directly extrapolated to other exposure scenarios. Second, our observations are confined to the acute phase post-irradiation. The long-term trajectory of proton-induced chronic cognitive impairment, and whether mitophagy dysfunction persists or evolves as a driving mechanism of delayed neurodegeneration, remains to be elucidated in longitudinal studies. Third, our current data provide correlative evidence linking BNIP3-associated mitophagy dysregulation to proton-induced neuronal damage and cognitive deficits, but causal validation via genetic manipulation is still lacking. Future studies using hippocampal neuron-specific conditional knockout of BNIP3 or Ulk1 will be required to definitively establish the causal role of mitophagy in proton radiation-induced cognitive injury. From a translational perspective, the development of small-molecule modulators that precisely tune mitophagic flux, combined with mitochondria-targeted antioxidants, represents a promising strategy to improve radioprotective efficacy against central nervous system injury.

## Figures and Tables

**Table 1:** Key chemical reagents used in this study.

Reagent or Resource	Source	Identifier
**Antibodies**		
Mouse Recombinant Anti-LC3B antibody	Abcam (Cambridge, UK)	Cat# ab192890, RRID:AB_2827794
Recombinant Anti-TOM20 antibody	Abcam	Cat# ab186735, RRID:AB_2889972
BNIP3 antibody	Abcam	Cat# ab109362, RRID:AB_10864714
Phospho-ULK1 (Ser757) Antibody	Cell Signaling Technology (Cambridge, UK)	Cat# 6888S
Recombinant Anti-Cytochrome C antibody	Abcam	Cat# ab133504, RRID:AB_2802115
Recombinant Anti-SQSTM1/p62 antibody	Abcam	Cat# ab109012, RRID:AB_2810880
AMPK alpha 1 antibody	Abcam	Cat# ab32047, RRID:AB_722764
Anti-ATG12 antibody	Abcam	Cat# ab155589
LC3B (E5Q2K) Mouse Monoclonal Antibody	Cell Signaling Technology	Cat# 83506, RRID:AB_2800018
Goat Anti-Rabbit IgG H&L (HRP)	Abcam	Cat# ab6721, RRID:AB_955447
Goat Anti-Rabbit IgG H&L (Alexa Fluor^®^ 488)	Abcam	Cat# ab150077, RRID:AB_2630356
Goat Anti-Mouse IgG H&L (Cy3 ^®^)	Abcam	Cat# ab97035, RRID:AB_10680176
**Chemicals, peptides, and recombinant proteins**		
Mito-TEMPO	Merck (Darmstadt, Germany)	SML0737-25MG
DMEM (high glucose)	Procell (Wuhan, China)	PM150210
HT22 Cell Complete Medium	Procell	CM-0697
DMSO	Beyotime (Shanghai, China)	ST038
DAPI	Beyotime	C1002
Hoechst 33342	Beyotime	C1028
MitoTracker™ Green FM	Thermo Fisher Scientific (Waltham, MA, USA)	M7514
LysoTracker Red DND-99	Thermo Fisher Scientific	L7528
Nissl Staining Solution	Thermo Fisher Scientific	N21479
**Critical commercial assays**		
8-OHdG ELISA Kit	Abcam	ab285254
Mouse IL-1β ELISA Kit	Abcam	ab197742
Mouse IL-4 ELISA Kit	Abcam	ab100710
Mouse IL-6 ELISA Kit	Abcam	ab222503
Mouse IL-10 ELISA Kit	Abcam	ab255729
Mouse IFN-γ ELISA Kit	Abcam	ab282874
MouseTNF-α ELISA Kit	Abcam	ab208348
GSH-Px Activity Assay Kit	Elabscience (Wuhan, China)	E-BC-K096-M
T-AOC Colorimetric Assay Kit	Elabscience	E-BC-K136-M
GABA ELISA Kit	Elabscience	E-BC-F048
5-HT ELISA Kit	Elabscience	E-EL-0033
ACHELISA Kit	Elabscience	E-EL-0081
8-OHdG ELISA Kit	Elabscience	E-EL-0028
Enhanced JC-1 Assay Kit	Beyotime	C2003S
Enhanced ATP Assay Kit	Beyotime	S0027
ROS Assay Kit	Beyotime	S0036M
SYBR Green qPCR Mix	Beyotime	D7265
RNA/Protein Isolation Kit	Beyotime	R0018M
cDNA synthesis kit	Beyotime	D7180M
Enhanced BCA Protein Assay Kit	Beyotime	P0010
Electrophoresis, Transfer and Western Blot Kit	Beyotime	P0002M
**Experimental models: Organisms/strains**		
C57BL-6J (male)	Nanjing Junke Bioengineering Co., Ltd. (Nanjing, China)	N/A
**Experimental models: Cell lines**		
HT22	Procell	CL-0697
**Oligonucleotides**		
All primers used in this study	Sangon (Shanghai, China)	Table 1
**Software and algorithms**		
Origin	OriginLab	v10.3
Edraw	Wondershare	V15.2
Image J	NIH	v1.51
Zen	Carl Zeiss	v7.0

**Table 2:** All primers used in this study.

Gene	Primer Orientation	Primer Sequence
AMPK	FORWARD	ACCCACAGAAATCCAAACACCAAG
AMPK	REVERSE	CAACCTTCCATTCATAATCCAACTGC
mTOR	FORWARD	GATGTGCCGAGACCTTGAGTTG
mTOR	REVERSE	GCCTCTGCTTGGATGTGATGAC
ATG12	FORWARD	GACCATCCAAGGACTCATTGACTTC
ATG12	REVERSE	GTCTGGGGAAGGGGCAAAGG
BNIP3	FORWARD	AACTCAGATTGGATATGGGATTGGTC
BNIP3	REVERSE	CCCTTTCTTCATAACGCTTGTGTTTC
TOM20	FORWARD	CCACCAGTGTTCCAGATGCT
TOM20	REVERSE	TCTTCAGCCAAGCTCTGAGC
LC3B	FORWARD	CTGAGCAGAGGGAAAAGGGG
LC3B	REVERSE	CCAGGAGGAAGAAGGCTTGG
P62	FORWARD	CTGCTCTTCGGAAGTCAGCA
P62	REVERSE	ATCGTCTCCTCCTGAGCAGT
ULK1	FORWARD	CTTCAGCACCAGCCGCATTAC
ULK1	REVERSE	AGCCAGCAGAGGGAGCAATC
Cytochrome C	FORWARD	TCACCTGGGGAGAGGATACC
Cytochrome C	REVERSE	GGTCTGCCCTTTCTCCCTTC
GAPDH	FORWARD	TGATGGGTGTGAACCACGAG
GAPDH	REVERSE	AGTGATGGCATGGACTGTGG

## Data Availability

The original contributions presented in this study are included in the article/Supplementary Material. Further information and requests for materials should be directed to and will be fulfilled by the lead contact, Longzhen Zhang (23B936149@stu.hit.edu.cn). Raw data and unique cell lines are available by request from the corresponding author, Weihong Lu (lwh@ hit.edu.cn).

## Supplementary Materials

The following supporting information can be downloaded at: https://www.mdpi.com/article/10.3390/biom16091342/s1, Video S1: Continuous real-time imaging of the Ctrl group; Video S2: Continuous real-time imaging of the RAD group. File S1: Original Western Blot images.
